# MnGa-based fully perpendicular magnetic tunnel junctions with ultrathin Co_2_MnSi interlayers

**DOI:** 10.1038/srep43064

**Published:** 2017-02-24

**Authors:** Siwei Mao, Jun Lu, Xupeng Zhao, Xiaolei Wang, Dahai Wei, Jian Liu, Jianbai Xia, Jianhua Zhao

**Affiliations:** 1 State Key Laboratory of Superlattices and Microstructures, Institute of Semiconductors, Chinese Academy of Sciences, P.O. Box 912,Beijing 100083, China

## Abstract

Because tetragonal structured MnGa alloy has intrinsic (not interface induced) giant perpendicular magnetic anisotropy (PMA), ultra-low damping constant and high spin polarization, it is predicted to be a kind of suitable magnetic electrode candidate in the perpendicular magnetic tunnel junction (p-MTJ) for high density spin transfer torque magnetic random access memory (STT-MRAM) applications. However, p-MTJs with both bottom and top MnGa electrodes have not been achieved yet, since high quality perpendicular magnetic MnGa films can hardly be obtained on the MgO barrier due to large lattice mismatch and surface energy difference between them. Here, a MnGa-based fully p-MTJ with the structure of MnGa/Co_2_MnSi/MgO/Co_2_MnSi/MnGa is investigated. As a result, the multilayer is with high crystalline quality, and both the top and bottom MnGa electrodes show well PMA. Meanwhile, a distinct tunneling magnetoresistance (TMR) ratio of 65% at 10 K is achieved. Ultrathin Co_2_MnSi films are used to optimize the interface quality between MnGa and MgO barrier. A strong antiferromagnetic coupling in MnGa/Co_2_MnSi bilayer is confirmed with the interfacial exchange coupling constant of −5erg/cm^2^. This work proposes a novel p-MTJ structure for the future STT-MRAM progress.

The spin transfer torque magnetic random access memory (STT-MRAM) is moving from an emerging technology to a main-stream one because it is a promising candidate for embedded memory combining low power consumption, high speed performance, non-volatility, high storage density, high thermal stability and practically unlimited read and write endurance. These advantages of MRAM make severe demands on magnetic tunnel junctions (MTJs) with perpendicular magnetic anisotropy (PMA) electrode materials^1^,^2^,^3^. In the past several decades, scientists have made great progress in the research about interface induced PMA effect existing in the material systems such as CoFeB/MgO and Co-based multilayers [Co/Pt, Pd]_n_^4^,^5^. However, such interfacial PMA materials have notable disadvantages. Take CoFeB/MgO for example, the PMA of CoFeB is very sensitive to the film thickness and buffer layer types^4^, i.e., a precisely controlled material preparation is necessary. In addition, the damping constant of CoFeB increases rapidly as its thickness decreases^6^,^7^, which may cause high STT switching current density. In the case of [Co/Pt, Pd]_n_, its large damping constant doesn’t support low power current-induced switching^8^ and the introduction of noble metal moreover limits its wide application. To overcome these difficulties, novel electrode material systems with intrinsic bulk PMA and low damping constant should be exploited to replace the interface induced PMA materials.

Among all the candidates of PMA materials, tetragonal structured MnGa alloy shows unique superiorities. An intrinsic bulk perpendicular magnetocrystalline anisotropy (*K*_u_) of MnGa up to 21.7 Merg/cc was achieved^9^, which is large enough to meet the requirement of stability for MTJ technology at sub-10 nm nodes. The saturated magnetization (*M*_s_) of MnGa can be tuned from 27.3 to 270.5 emu/cc by changing component and growth condition^9^, so that it is suitable for both the free-layer and pinning layer in STT-MRAM devices. In addition, its damping constant is ultralow as 0.0003 from the theoretical prediction based on band calculation^10^, which is a key factor for low power consumption. Moreover, its high spin polarization of 88%^11^ (in theory) as well as high Curie temperature of 630 K^12^ are also appropriate for high tunneling magnetoresistance (TMR) ratio and high thermal stability. Recently, MnGa have been investigated for MgO-based MTJs and shown excellent TMR effect theoretically^13^, but the prospective high performance device has not been realized experimentally yet^14^,^15^,^16^,^17^. In the preliminary works of Miyazaki’s group, they reported MnGa-based MTJs with a sensor structure of MnGa/MgO/CoFe and the TMR ratio was observed to be 23% at 10 K^15^. Then they tried to fabricate perpendicular magnetic tunnel junction (p-MTJ) structures such as MnGa/FM/MgO/CoFeB with TMR ratio up to 50% at room temperature^16^,^17^. However, the works mentioned above chose MnGa for only bottom electrodes in the MTJs so that they could not take full advantage of the superiority of MnGa. To our best knowledge, experimental results of all bulk MnGa-based p-MTJs (like MnGa/MgO/MnGa) have not been reported yet, since high quality MnGa films with giant PMA are difficult to obtain directly on MgO barrier.

In this work, we present the realization of all bulk MnGa-based fully p-MTJs with the core structure of Mn_3.1_Ga(23 nm)/Co_2_MnSi(0.6 nm)/MgO(1.8 nm)/Co_2_MnSi(0.6 nm)/Mn_2.9_Ga(12 nm) (from the bottom to top). The whole structure is grown on GaAs (001) substrates by molecular-beam epitaxy (MBE) system with two chambers (VG80) without being exposed to the air during the entire process. As a result, the multilayer have a high-quality crystalline structure and a distinct TMR effect (65% at 10 K) is achieved. Here, we choose ultrathin half-metallic Heusler compound Co_2_MnSi films as interlayers to reduce the lattice mismatch between MgO barrier and MnGa electrodes as well as to enhance the TMR ratio. Simulated results show strong antiferromagnetic (AFM) coupling in MnGa/Co_2_MnSi bilayer with an interfacial coupling constant (*J*_ex_) of −5.0 erg/cm^2^, so that the magnetic moment rotation of Co_2_MnSi interlayers can be effectively controlled.

## Results and Discussion

### Antiferromagnetic exchange coupling between MnGa and Co_2_MnSi

The AFM exchange coupling between Co-based Heusler alloys and MnGa films has been investigated previously by Ranjbar *et al*.^18^ Among several kinds of Co-based Heusler compounds, Co_2_MnSi was identified to show the highest interfacial AFM coupling strength (*J*_ex_) with MnGa^19^.

**Figure 1:**
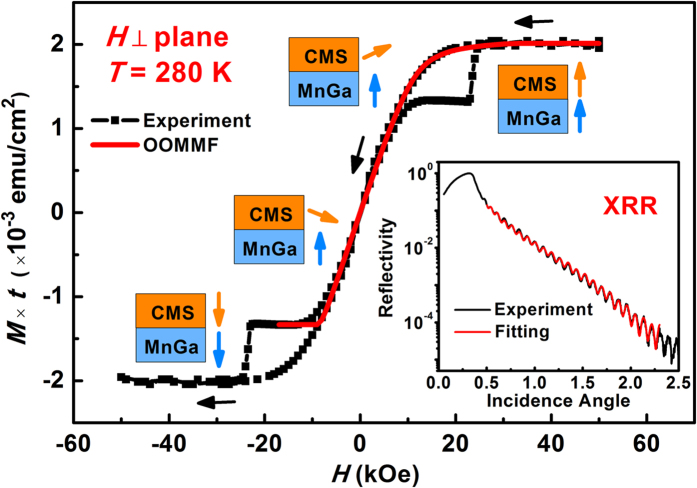
Out-of-plane hysteresis loop of Co_2_MnSi/MnGa bilayer measured at 280 K (black scattered line) and OOMMF simulated loop with *J*_ex_ = −5 erg/cm^2^ (red line). The inset shows the XRR data of the bilayer.

Figure 1 shows the out-of-plane hysteresis loop of Co_2_MnSi(20 nm)/MnGa(28 nm) bilayer epitaxially grown on GaAs (001) substrate. The film thickness is confirmed by x-ray reflectivity (shown in the inset). As the magnetic field scanning from 50 kOe to −50 kOe, the magnetic moment of Co_2_MnSi starts to rotate from positive orientation to negative one under the impact of both external field and AFM exchange coupling field from MnGa layer. Meanwhile, the sharply jump at high field region originates from the switch of MnGa magnetization. The interfacial coupling constant *J*_ex_ of the bilayer can be fitted by micromagnetic simulation software object oriented micromagnetic framework (OOMMF), and the total interfacial exchange energy density can be defined by the following relation^20^:


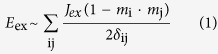


where i and j refer to the matching cell at each side of the interface; *m*_i_ and *m*_j_ are normalized unit spin (magnetization directions) at cell i and j; *δ*_ij_ is the discretization cell size. Our sample was simulated with a mesh size of 4 × 4 × 1 nm. The calculation parameters of saturated magnetization 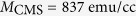 and 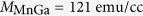 were taken from our experimental values (See Supplementary Section 1). The exchange stiffness constants 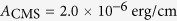
21, 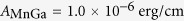 were used, while the magnetocrystalline anisotropy parameters were 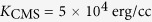 (cubic)^22^ and 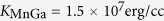 (uniaxial)^9^. As a result, the simulation curve with 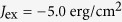 fitted with experimental data very well, as shown in Fig. 1. It suggests a very strong AFM coupling at the Co_2_MnSi/MnGa interface, therefore, the moment orientation of the two layers can be modulated by each other effectively.

### MTJ stacking and crystal structures

Motivated by the strong AFM coupling between Co_2_MnSi/MnGa bilayer, we chose a Co_2_MnSi film with the thickness of 0.6 nm (the thickness was chosen optimally) as interlayer between MnGa electrodes and MgO barrier to fabricate MnGa-based fully p-MTJ. Meanwhile, the lattice mismatch between MgO/Co_2_MnSi (bulk) is only 5%, compared to that of 7.7% at MgO/MnGa interface.

**Figure 2:**
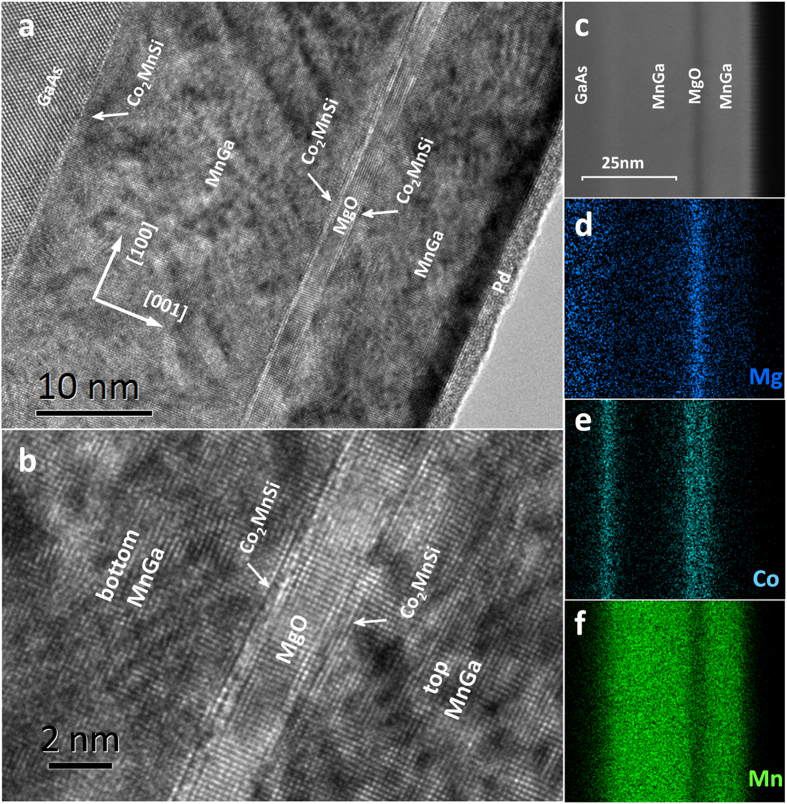
(**a**) HRTEM image of the whole MTJ structure. (**b**) HRTEM image of the region around MgO barrier. (**c**) Z-contrast STEM image. (**d**–**f**) Plane-scan EDS data of Mg, Co and Mn element distributions.

Cross-section high-resolution transmission electron microscopy (HRTEM) images were taken to show the interfacial details of our MTJ structure. As shown in Fig. 2a and b, the multilayers with the core structure of Mn_3.1_Ga(23 nm)/Co_2_MnSi(0.6 nm)/MgO(1.8 nm)/Co_2_MnSi(0.6 nm)/Mn_2.9_Ga(12 nm) (from the bottom to top) are epitaxially grown along the (001) orientation. Both the top Co_2_MnSi and the bottom Co_2_MnSi interlayers can be clearly identified (shown in Fig. 2a). Specifically, from Fig. 2b, one can see that not only the bottom MnGa but also the top MnGa layers are with good-quality single-crystalline structure. (The tetragonal structure of MnGa can be proved by XRD pattern, see Supplementary Section 2) However, it should be noted that, the bottom Co_2_MnSi/MgO interface is much sharper than the top one under the same contrast (shown in Fig. 2a). The lattice mismatch as well as large different surface energy between MgO and the top metal interlayer lead to the three-dimensional (3D) growth mode of top Co_2_MnSi as indicated by the *in-situ* RHEED patterns^23^, which degrades the interfacial flatness so that the quality of top Co_2_MnSi/MgO interface is not so high as the bottom one. Figure 2c shows the Z-contrast STEM image of the sample and Fig. 2d–f are plane-scan energy dispersive spectroscopy (EDS) results of Mg, Co and Mn elements respectively. The element distribution in the multilayer is clear while the MgO layer plays an important role as a diffusion barrier for the metal atoms.

### Magnetic and transport properties

**Figure 3:**
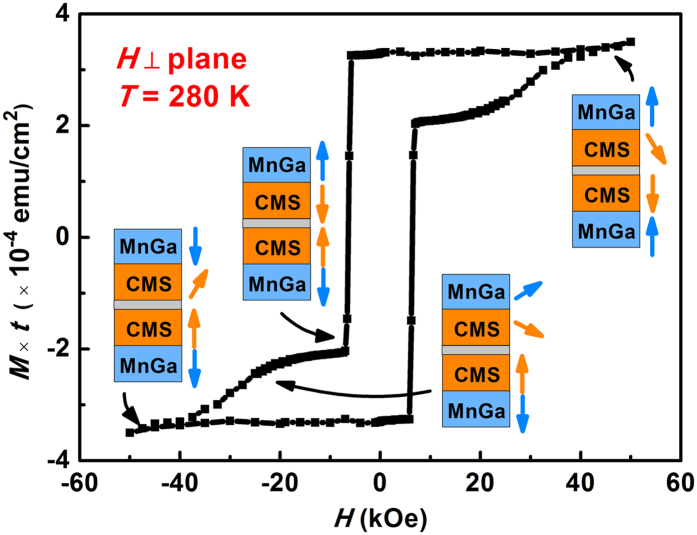
Hysteresis loop of the MTJ sample measured at 280 K with magnetic field perpendicular to the sample surface.

Figure 3 shows the hysteresis loop of our structure carried out at 280 K. After an initial magnetization process under the magnetic field of 50 kOe, the moment of MnGa orients parallel to external field whereas Co_2_MnSi is AFM coupled with it simultaneously. The sharp drop in the hysteresis loop at about −6 kOe results from the magnetic reversal of the bottom MnGa electrode, while the gradually change at the range of −20 kOe ~−40 kOe is caused by the moment rotation of top MnGa electrode. It has been reported previously that high quality of epitaxial MnGa depends highly on the type of substrates and even very sensitive to the reconstruction at the surface^24^,^25^,^26^. After the room temperature growth of MgO barrier and low temperature annealing process, the surface of our multilayer can hardly maintain flat at atomic scale, which increases the chemical disorder of the subsequent grown MnGa layer. It’s an important reason for the large *H*_c_ and broad shape of the hysteresis loop of the top MnGa electrode.

**Figure 4:**
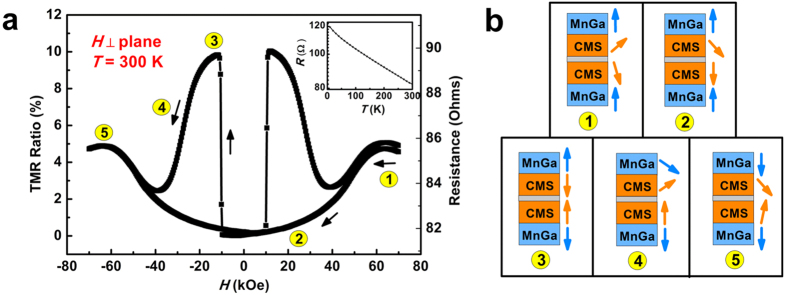
(**a**) Room-temperature TMR behavior for MnGa/Co_2_MnSi/MgO/Co_2_MnSi/MnGa structure. The inset shows the temperature dependence of the junction resistance. (**b**) Schematic diagram of the magnetization state.

Figure 4a shows the *R-H* curve of the device measured at room temperature (300 K). To clarify the magnetoresistance behavior of our MTJ sample, a maximum field of ±70 kOe was applied. As the external field scanning from +70 kOe to −70 kOe, the magnetization state can be concluded into five stages (shown in Fig. 4b), which will be discussed in detail as following. In stage 1, the external field is set to 70 kOe, however, the junction resistance doesn’t perform at the minimum value as expected. This can be explained by a partly destruction of the AFM coupling between the top MnGa layer and Co_2_MnSi interlayer where the high external field plays a dominating role. As mentioned above, the chemical order in the top MnGa is not very high, which may decrease the AFM coupling strength of top Co_2_MnSi/MnGa bilayer. Although the effective AFM coupling field (*H*_eff_) of bottom MnGa/Co_2_MnSi interface is larger than 60 kOe (estimated by the formula 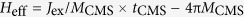), the AFM coupling of top MnGa/Co_2_MnSi is not completely preserved under such a high field. So that the moment of two Co_2_MnSi interlayers at each side of MgO barrier can hardly orient parallel to each other. As the external field decreases, the AFM coupling of bilayer tends to dominate. The approximate parallel moment arrangement of two interlayers leads to the decrease of junction resistance in stage 2. In stage 3, the negative field further increases to the coercivity of bottom MnGa electrode. The moment of bottom Co_2_MnSi interlayer is reversed with bottom MnGa simultaneously, hence the junction resistance jumps to a high level. In stage 4, the moment of top MnGa rotates with the external field (which is coincident with the hysteresis loop) and the two interlayers tend to be parallel oriented as a result of the competition between external field and the exchange coupling field. Finally, the effect of negative external field dominates once again in stage 5, where the moment of the multilayer turns to a reverse state of stage 1.

**Figure 5:**
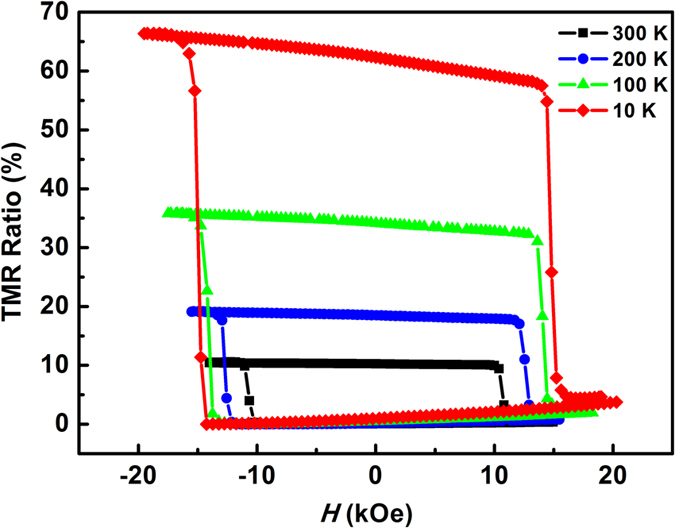
Temperature dependence of TMR ratio for MnGa-based MTJ. (Minor loop within ±20 kOe).

Figure 5 shows the relationship between TMR ratio and ambient temperature. TMR ratio calculated by 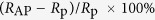 are 10% at 300 K and 65% at 10 K, respectively. The strong temperature dependence of the TMR behavior is mostly attributed to a spin-flip tunneling process. Interfacial diffusion of Mn or Ga atoms may bring magnetic impurities into the MgO barrier (the quality of MgO barrier can be evaluated by fitting the *I-V* relationship^27^, see Supplementary Section 3), which causes the scattering mechanism to flip the spin and suppress the TMR effect while increasing ambient temperature^28^.

**Figure 6:**
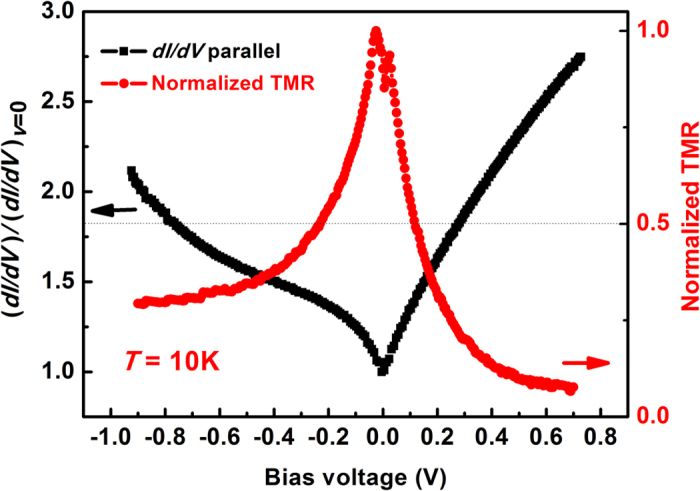
Bias voltage dependence of (*dI*/*dV*)/(*dI*/*dV*)_*V*=0_ in the parallel magnetization configuration (black line) and bias voltage dependence of TMR ratio (red line) measured at 10 K.

Furthermore, in order to better understand the TMR behavior of our devices, bias voltage dependence of differential-conductance ((*dI*/*dV*)/(*dI*/*dV*)_*V*=0_) (black line) and normalized TMR ratio (red line) was measured using current source (mode 6221, KEITHLEY Inst. Inc.) and nanovoltmeter (mode 2182, KEITHLEY Inst. Inc.), as shown in Fig. 6. In this figure, the positive bias region corresponds to electrons tunneling from bottom Co_2_MnSi interlayer into top Co_2_MnSi interlayer. A shoulder-structure at nearly −0.4 V of *dI*/*dV* is observed, which is related to the coherent tunneling process through MgO barrier^29^,^30^. However, similar features are not apparent at the positive bias region. Such asymmetric bias dependence has been observed in a lot of junctions such as Fe/MgO/Fe and CoFeB/MgO/CoFeB, as the sample structure is stoichiometrically symmetric, the origin of asymmetric transport behavior can be attributed to dissimilar interfacial states at each side of MgO barrier^31^,^32^,^33^. While the MgO barrier is epitaxial grown on a Co_2_MnSi layer with atomic flatness, the top Co_2_MnSi insert is grown on the MgO surface with higher roughness (as indicated by the RHEED pattern *in situ* observed during growth). Different growth conditions lead to various interfacial defect density and even different degree of interfacial oxidation, then the different interfacial states further cause a asymmetric transport property. It’s worth noting that the sharp drop of TMR*-V* curve reflects the quality of barrier/interlayer interface not very high^34^. Room temperature growth of the MgO and a low temperature annealing process cause a non-atomic flatness barrier surface, which is conductive to the appearance of dislocations and vacancies. Besides, the lattice mismatch also contributes to the generation of interfacial defects. All of these will make bad influence to the electron tunneling procedure. So the mediocre TMR ratio of our MTJ may be mainly from the interface defects, especially at the top Co_2_MnSi/MgO interface. This problem might be eliminated by optimizing the annealing process or using other suitable interlayers.

In conclusion, we have investigated firstly TMR effect of the MnGa-based fully perpendicular MTJ with Co_2_MnSi Heusler alloy interlayers. The strong interfacial AFM coupling between Co_2_MnSi/MnGa has been verified and the moment rotation of Co_2_MnSi interlayer could be manipulated in our MTJ structure. The magnetization process of the MTJ has been analyzed carefully and a TMR ratio up to 65% at 10 K (10% at 300 K) has been achieved. HRTEM images as well as bias voltage dependence of differential-conductance have been carried out to explain the mediocre tunneling behavior, which might be caused by the structure defects at the barrier/interlayer interfaces. This work takes full advantage of the intrinsic PMA of MnGa and proposes a novel structure of MnGa-based p-MTJ for future MRAM applications.

## Method

### Sample preparation

Two samples with stacking structure of Co_2_MnSi(20 nm)/MnGa(28 nm) and Co_2_MnSi buffer(0.6 nm)/Mn_3.1_Ga(23 nm)/Co_2_MnSi(0.6 nm)/MgO (1.8 nm)/Co_2_MnSi (0.6 nm) /Mn_2.9_Ga (12 nm) (from the bottom to top) were prepared by an MBE (VG80) system with two chambers on GaAs (001) substrate without being exposed to the air during the entire process. The metallic films were grown at 250–300 °C while the MgO barrier was deposited with e-beam evaporation at room temperature respectively. After growth, the films were subsequently annealed at 300 °C for 20 minutes and covered by a 2 nm Pd capping layer for protection. The whole process was monitored *in-situ* by reflection high-energy electron diffraction (RHEED).

### Sample characterization

The crystalline structures were characterized by *ex-situ* cross-sectional high-resolution transmission electron microscopy (HRTEM JEOL 2010 F) and the element content of the films was measured by energy dispersive spectroscopy (EDS). Magnetic properties of the multi-layers were characterized by quantum design superconducting quantum interference device (SQUID) with a maximum applied field of ±50 kOe at 280 K. MTJ devices were fabricated into 50 × 50 μm^2^ junctions by using UV lithography and Ar ion beam etching techniques. SiO_2_ and Cr/Au were used for insulating and connecting electrode materials. The transport behavior was measured by quantum design physical property measurement system (PPMS) using four-terminal method.

## Additional Information

**How to cite this article**: Mao, S. *et al*. MnGa-based fully perpendicular magnetic tunnel junctions with ultrathin Co_2_MnSi interlayers. *Sci. Rep.* **7**, 43064; doi: 10.1038/srep43064 (2017).

**Publisher's note:** Springer Nature remains neutral with regard to jurisdictional claims in published maps and institutional affiliations.

## Supplementary Material

Supplementary Information
