# Supplementary material for: MnGa-based fully perpendicular magnetic tunnel junctions with ultrathin Co2MnSi interlayers
Source: Sci Rep. 2017 Feb 24;7:43064. doi: 10.1038/srep43064 (PMC5324047; doi:10.1038/srep43064)
Supplement: Supplementary Information [file srep43064-s1.pdf]

Supplementary Information

MnGa-based fully perpendicular magnetic tunnel junctions with ultrathin Co_2MnSi interlayers

Siwei Mao, Jun Lu*, Xupeng Zhao, Xiaolei Wang, Dahai Wei, Jian Liu, Jianbai Xia & Jianhua Zhao*

State Key Laboratory of Superlattices and Microstructures, Institute of Semiconductors, Chinese Academy of Sciences, P.O. Box 912,Beijing 100083, China.

*Correspondence should be addressed to:

J. L. (email: lujun@semi.ac.cn) or J. H. Z. (email: jhzhao@red.semi.ac.cn)

Supplementary Information

Section 1: The saturated magnetization of MnGa and Co_2MnSi layers in our experiment.

The saturated magnetization of Co_2MnSi and MnGa layers are also obtained from the hysteresis loop of our MnGa/Co_2MnSi bilayer samples, which is shown particularly in **Figure S1**. As external magnetic field scanning from 50 kOe to -50 kOe, the moment change Δm_1 and Δm_2 (see **Figure S1**) reflect the saturated moments of Co_2MnSi and MnGa, respectively. Since $\Delta m_1 = 3.99 \times 10^{-4}$emu, $\Delta m_2 = 8.10 \times 10^{-5}$emu, the sample area used for SQUID measurement is $s = 11.92\ \text{mm}^2$ (3.04 mm× 3.92 mm in rectangular shape, confirmed by vernier caliper), film thickness are $t_{\text{MnGa}} = 28\ \text{nm}$, t_{CMS} =20 nm (confirmed by XRR measurement), the saturated magnetization of Co_2MnSi and MnGa can be defined by the following relations:

$$M_{\text{CMS}} = \frac{m_{\text{CMS}}}{\text{V}_{\text{CMS}}} = \frac{\frac{\Delta m_1}{2}}{s \times t_{\text{CMS}}} = 837\ \text{emu/cc}$$

$$M_{\text{MnGa}} = \frac{m_{\text{MnGa}}}{\text{V}_{\text{MnGa}}} = \frac{\frac{\Delta m_2}{2}}{s \times t_{\text{MnGa}}} = 121\ \text{emu/cc}$$

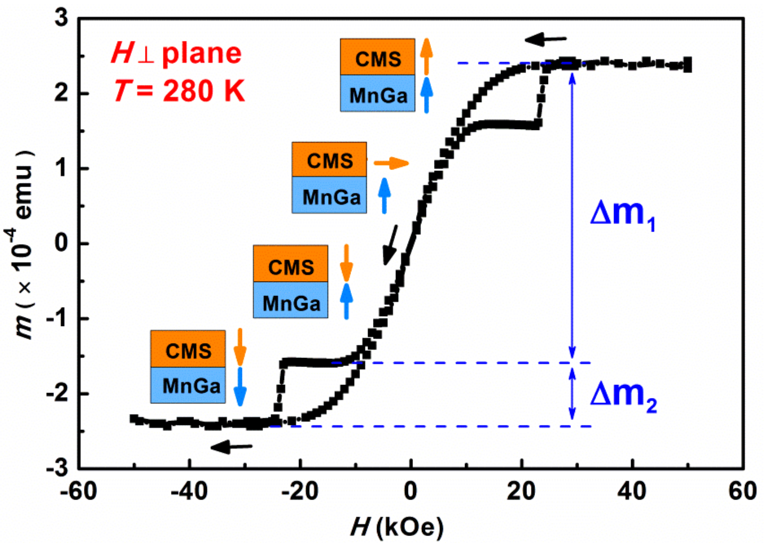

Figure S1. Hysteresis loop of the bilayer sample.

Section 2: Evidence for the tetragonal structure of MnGa electrodes.

Figure S2 shows the XRD θ-2θ pattern of our MTJ structure measured along the [001] direction. In addition to peaks from GaAs substrate and Pd cap, (002) and (004) peaks of tetragonal MnGa are separately highlighted in the figure, which suggests that single phase tetragonal MnGa electrodes are obtained in the sample.

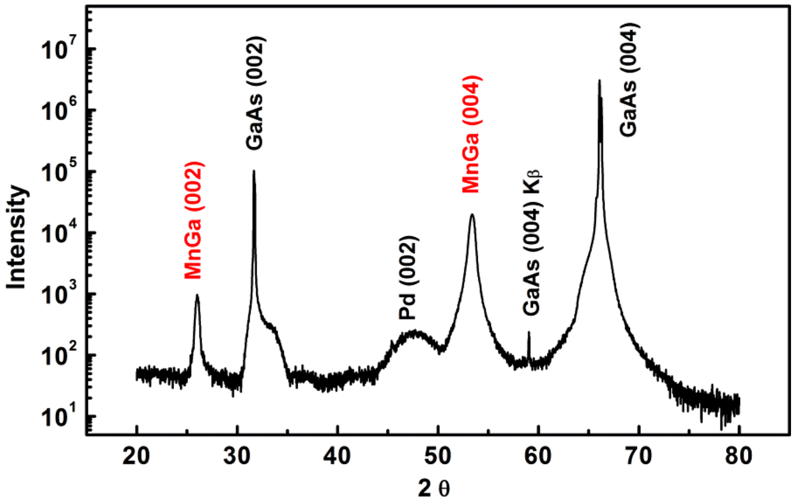

Figure S2. XRD data of the MTJ sample.

Section 3: Evaluation of the quality of MgO barrier.

As the MgO barrier in our multilayer is ultrathin (~2 nm), to directly analyze its crystal structure and atom component is a quite challenging work. Nevertheless, we can evaluate the barrier's quality indirectly by measuring the *I-V* curve of our MTJ sample (shown in Figure S3), which reveals the information of the atom diffusion degree.

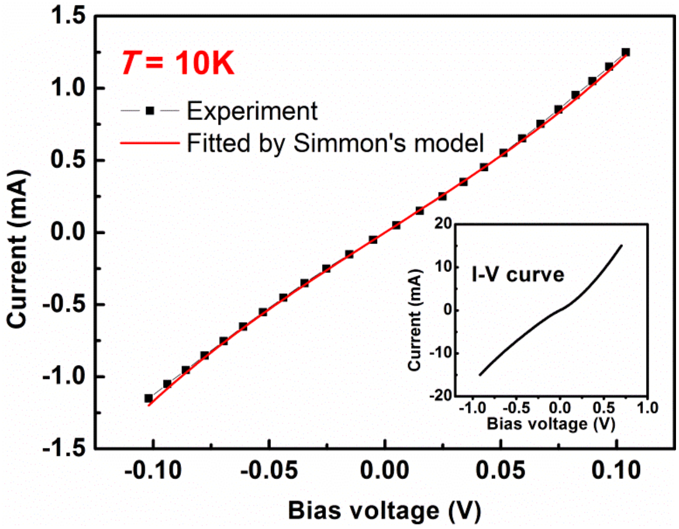

Figure S3. *I-V* curve measured by current source (mode 6221, KEITHLEY Inst. Inc.) and nanovoltmeter (mode 2182, KEITHLEY Inst. Inc.) The red line shows the result fitted by Simmon's model. Wide range *I-V* relationship is shown in the insert, a nonlinear *I-V* behavior reflects the character of tunneling conductance.

As shown in **Figure S3**, we fit the *I*-*V* characteristic by Simmon's model (suppose a rectangular potential barrier with bias voltage $V < \frac{\varphi}{e}$):

$$I = \frac{eA}{2\pi h t^2}\{(\varphi - \frac{eV}{2}) \exp[\frac{-4\pi t}{h}(2m)^{\frac{1}{2}}\left(\varphi - \frac{eV}{2}\right)^{\frac{1}{2}}] - (\varphi + \frac{eV}{2})\exp[\frac{-4\pi t}{h}(2m)^{\frac{1}{2}}\left(\varphi + \frac{eV}{2}\right)^{\frac{1}{2}}]\}$$

Here, I is the current, A is the junction area ($50 \times 50\mathrm{\mu m}^2$ in our case), e is electron charge, h is plank constant, t is the effective barrier thickness and φ is the effective barrier height. We limited the voltage range of our fitting within $\pm 0.1\,V$, for the non-negligible heating effect caused by high current may interfere the measurement. As a result, the fitted value shows $t \sim 2.2\,\mathrm{nm}$ and $\varphi \sim 0.39\,eV$. Since the fitted barrier thickness is close to our experimental parameter, the effective barrier height is a bit low. It's an evidence to support the influence of impurity diffusion into MgO barrier.
